# Role of Human Sec63 in Modulating the Steady-State Levels of Multi-Spanning Membrane Proteins

**DOI:** 10.1371/journal.pone.0049243

**Published:** 2012-11-15

**Authors:** Andreas Mades, Katherina Gotthardt, Karin Awe, Jens Stieler, Tatjana Döring, Sabine Füser, Reinhild Prange

**Affiliations:** 1 Department of Medicine III, Hematology and Oncology, Johannes Gutenberg-University School of Medicine, Mainz, Germany; 2 Department of Medical Microbiology and Hygiene, University Medical Center of the Johannes Gutenberg University Mainz, Mainz, Germany; Ecole Polytechnique Federale de Lausanne, Switzerland

## Abstract

The Sec61 translocon of the endoplasmic reticulum (ER) membrane forms an aqueous pore, allowing polypeptides to be transferred across or integrated into membranes. Protein translocation into the ER can occur co- and posttranslationally. In yeast, posttranslational translocation involves the heptameric translocase complex including its Sec62p and Sec63p subunits. The mammalian ER membrane contains orthologs of yeast Sec62p and Sec63p, but their function is poorly understood. Here, we analyzed the effects of excess and deficit Sec63 on various ER cargoes using human cell culture systems. The overexpression of Sec63 reduces the steady-state levels of viral and cellular multi-spanning membrane proteins in a cotranslational mode, while soluble and single-spanning ER reporters are not affected. Consistent with this, the knock-down of Sec63 increases the steady-state pools of polytopic ER proteins, suggesting a substrate-specific and regulatory function of Sec63 in ER import. Overexpressed Sec63 exerts its down-regulating activity on polytopic protein levels independent of its Sec62-interacting motif, indicating that it may not act in conjunction with Sec62 in human cells. The specific action of Sec63 is further sustained by our observations that the up-regulation of either Sec62 or two other ER proteins with lumenal J domains, like ERdj1 and ERdj4, does not compromise the steady-state level of a multi-spanning membrane reporter. A J domain-specific mutation of Sec63, proposed to weaken its interaction with the ER resident BiP chaperone, reduces the down-regulating capacity of excess Sec63, suggesting an involvement of BiP in this process. Together, these results suggest that Sec63 may perform a substrate-selective quantity control function during cotranslational ER import.

## Introduction

The ER is a major site of synthesis for both secretory and membrane integrated proteins. Generally, nascent polypeptides are translocated cotranslationally across or into the ER membrane at sites termed translocons. The translocon minimally comprises one Sec61α-β-γ heterotrimer and associated proteins forming an aqueous conduit that aligns with the ribosome during translocation. In cotranslational translocation, signal sequences or transmembrane (TM) domains within a nascent polypeptide are recognized by the signal-recognition particle (SRP). The SRP-bound ribosome nascent chain complex then binds to the membrane via the SRP receptor, and after SRP release the translating ribosome interacts with the translocon, thereby enabling the growing polypeptide to be inserted directly into the translocon channel. Soluble proteins, such as those ultimately secreted from the cell or localized inside the ER lumen, cross the membrane completely, while membrane proteins exit laterally into the lipid phase, a step referred to as membrane integration [1], [2], [3]. The events accompanying the gating of the lateral exit portal of the translocon are currently less resolved.

Numerous components near the mammalian Sec61 core translocon complex have been described that could contribute to the overall efficiency of translocation. These include the SPC (signal peptidase complex) [4], OST (oligosaccharyl transferase complex) [5], TRAM (translocation chain-associated membrane protein) [6], [7], TRAP (translocon-associated protein) [8], RAMP4 (ribosome-associated membrane protein 4) [6], ER proteins with J domains inside the ER lumen such as ERdj1 and ERdj2/Sec63 [9], [10], [11], the Sec63-interacting Sec62 protein [10], and others. The functional roles of the translocon-associated Sec62 and Sec63 proteins in protein biogenesis at the ER have been well established in the yeast *Saccaromyces cerevisiae*, where nascent chains can be translocated either co- or posttranslationally [1], [2], [3]. Both translocation pathways depend on the protein-conducting Sec61 channel, but differ in their targeting mechanisms. In the cotranslational mode of translocation, nascent precursors are targeted in an SRP-dependent manner. In the posttranslational mode of translocation, the Sec61 channel partners with another membrane protein complex, consisting of the essential Sec62p and Sec63p proteins, and the non-essential Sec71p and Sec72p proteins, that together mediate targeting and translocation of completed polypeptide chains. The posttranslational pathway is used predominantly by precursors with low hydrophobic signal peptides [1], [2], [12].

In the mammalian ER, the cotranslational mode of protein translocation dominates. Even so, orthologs of the yeast Sec62p and Sec63p, referred to as Sec62 and Sec63, are found in stoichiometric amounts compared with Sec61a and in association with the Sec61 complex [10], [13]. Sec62 is a double-spanning membrane protein with a cytosolic domain that associates with ribosomes, suggesting a cotranslational function of this protein in mammals [14]. Sec62 also teams up with Sec63, an integral membrane protein of the Hsp40 co-chaperone family [10], [14]. Similar to its yeast ortholog, mammalian Sec63 contains three TM domains with a lumenal J domain that functionally interacts with the ER resident BiP chaperone (Kar2p in yeast) [10], . The conservation of structural and biological features of Sec62 and Sec63 from yeast to humans strongly suggests that these proteins are likewise involved in protein transport into the mammalian ER, but their precise function is less known. Loss of function mutations in the human Sec63 are not lethal but are linked to polycystic liver disease, indicating that it may contribute to ER import and/or folding of proteins involved in biliary cell growth [1], [17]. In the case of human Sec62, its overexpression was found to be associated with sporadic colorectal cancer and prostate cancer [1]. The participation of Sec62/Sec63 in the biogenesis of particular ER substrates is also suggested by the finding that both proteins can transiently associate with newly synthesized polypeptides during ER membrane integration in cell-free systems [18].

Our previous work has been focused on the biogenesis of three related envelope proteins of the hepatitis B virus (HBV) that are synthesized as multi-spanning proteins at the ER membrane. While the small S and middle M envelope proteins are integrated into the ER membrane in a typical cotranslational process, the large L envelope protein forms a dual topology via a process involving cotranslational membrane integration and subsequent posttranslational translocation of its preS subdomain into the ER [19], [20], [21]. Similar to posttranslational translocation processes in yeast, the posttranslational preS translocation of L requires BiP [22], [23], a known interaction partner of Sec63 [10]. We took this as a rationale to study the function of human Sec63 and its partner proteins in cell culture systems. Against expectation, we find that Sec63 does not play a role in the special topogenesis of the HBV L protein (K.A and R.P., unpublished observation), but is rather more generally involved in the control of the steady-state levels of polytopic membrane proteins.

## Results

### Overexpressed Sec63, but not Sec62, Reduces the Steady-state Level of the Polytopic HBV.S Envelope Protein

**Figure 1:**
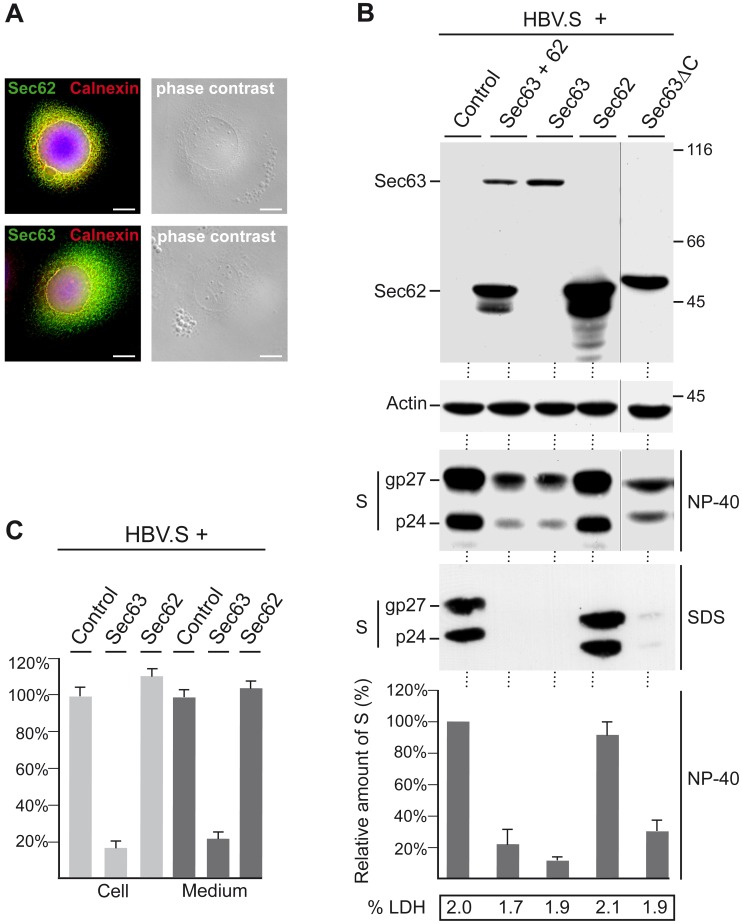
Overexpressing of Sec63 reduces the steady-state HBV.S level. A. HuH-7 cells were transfected with Myc-tagged Sec62 or Sec63 and immunostained with mouse anti-Myc and rabbit anti-calnexin antibodies. After staining with AlexaFluor 488-conjugated anti-mouse and AlexaFluor 546-conjugated anti-rabbit antibodies, cells were visualized by deconvolution fluorescence microscopy. The overlays of the fluorescences are shown with yellow colour indicating colocalization. DNA staining of the nuclei is in blue. The corresponding phase contrast images depicting nuclei and organelle structures in the cell periphery are shown in the right panels. Bar, 10 µm. **B.** Cells were transfected with HA-tagged HBV.S together with empty plasmid DNA (Control), Myc-tagged Sec62, Sec63, or Sec63ΔC at a 1∶3 DNA ratio (4 µg total DNA), respectively. Cotransfections of HBV.S with Sec62 plus Sec63 were done at a 1∶3∶3 DNA ratio (7 µg total DNA), respectively. Three days after transfection, cellular lysates were prepared with NP-40 (NP-40) and analyzed by HA- or Myc-specific Western blotting (WB) to demonstrate expression of HBV.S (bottom) or Sec62, Sec63 and Sec63ΔC (top). To confirm equal gel loading, the lysates were probed by anti-β-actin WB (middle top). Numbers to the right refer to molecular weight standards in kD, while the non-glycosyated (p24) and glycosylated (gp27) forms of HBV.S (S) are indicated on the left (middle bottom). Relative HBV.S expression values were determined by densitometric analysis and demonstrated below in the graph in percent amount relative to control cells. Error bars indicate the standard deviation from three separate experiments. The analyses of SDS lysates (SDS) of correspondingly cotransfected cells are illustrated in the bottom panel. To probe for cell cytotoxicity, supernatants of cotransfected cells were assessed for LDH activity. **C.** Cells were cotransfected with HBV.S and Sec62 or Sec63 exactly as in A. Lysates (Cell) and supernatants (Medium) were examined by an S-specific ELISA, and HBV.S levels are demonstrated in percent amount ± SD relative to control cells (*n* = 3).

To study the role of the human Sec62 and Sec63 proteins, expression vectors encoding N-terminally Myc-tagged versions of these proteins were constructed and transiently transfected into HuH-7 human liver carcinoma cells. Upon deconvolution immunofluorescence and phase contrast microscopy, the ectopically expressed Sec62 and Sec63 constructs yielded an ER-like staining and partially colocalized with calnexin, a membrane-anchored ER chaperone (Fig. 1A). The constructs were next transfected together with a C-terminally hemagglutinin (HA)-tagged version of the S envelope protein of HBV (HBV.S). HBV.S is cotranslationally integrated into the ER membrane, directed by the action of an uncleaved signal-anchor and a stop-transfer sequence encoded within its first and second TM segments [24]. The hydrophobic C-terminal half of HBV.S is predicted to further span the membrane two times thereby resulting in a fourfold membrane-spanning protein. After transient coexpression of the constructs, cell lysates were prepared with the non-denaturing detergent NP-40 and analyzed by Myc- and HA-specific Western blotting. As shown in Fig. 1B, Sec62 and Sec63 were efficiently synthesized and appeared in about 46 and 87 kDa forms, respectively, in accord with their calculated molecular masses. HBV.S was obtained in its characteristic doublet of a 24 kDa non-glycosylated (p24) and a 27 kDa glycosylated (gp27) form as a consequence of partial N-linked glycosylation [25]. Intriguingly, the intracellular level of HBV.S was significantly lower when ectopically expressed Sec63 was present. In contrast, excess Sec62 reproducibly did not affect the level of HBV.S (Fig. 1B). Similar results were obtained with cell lysates prepared with the denaturing detergent SDS (Fig. 1B), indicating that the observed down-regulation of HBV.S by excess Sec63 was not merely due to changes in the solubility profile. Since the overexpression of Sec63 might be harmful for the cells, cytotoxicity assays of cellular supernatants were performed which did not reveal significant indications of cell damage (Fig. 1B). Because Sec62 and Sec63 have been shown to associate with each other [13], [14], the up-regulation of Sec63 could evoke an imbalance of the complex partner. Therefore, the fate of HBV.S was analyzed in cells overexpressing both Sec62 and Sec63 proteins. Under these conditions, the levels of Sec62 and Sec63 were lower as compared to their individual expression. One explanation may be that Sec63 also reduced the steady-state level of the double-spanning Sec62 protein at the ER. Alternatively, this could be the consequence of a lower degree of transfection caused by the higher amount of plasmid DNA used for triple transfection. Nonetheless, even in consideration of a lower transfection rate, HBV.S was down-regulated (Fig. 1B), implicating that the steady-state level of Sec62 is less important. *In vitro*-binding studies have identified the negatively charged C-terminus of Sec63 as the interaction domain for basic oligopeptide motifs within the N-terminus of Sec62 [14]. To examine whether the Sec62/Sec63 interaction might play a role in the function of Sec63, we analyzed a C-terminally truncated Sec63 mutant lacking large parts of the cytosolic domain including the Sec62 binding site. This Sec63ΔC mutant similarly down-regulated HBV.S (Fig. 1B), thus arguing against an essential role of Sec62 in the observed action of Sec63.

Following ER translocation, the HBV.S protein is able to self-assemble into subviral envelope particles (SVPs) that bud into intralumenal cisternae of post-ER/pre-medial-Golgi compartments and exit the cell by the constitutive pathway of secretion [25], [26]. To investigate whether the intracellular reduction of HBV.S by overexpressed Sec63 was simply due to an enhanced SVP export, lysates and supernatants of cotransfected cells were examined by an HBV.S-specific ELISA. This analysis, however, clearly revealed that excess Sec63 reduced both the intra- and extracellular pool of HBV.S. In contrast, overexpressed Sec62 did not interfere with the synthesis and secretion of HBV.S (Fig. 1C).

### Overexpressed Sec63 Reduces the Steady-state Level of the Polytopic HBV.S Envelope Protein in a Dose-dependent Manner

**Figure 2:**
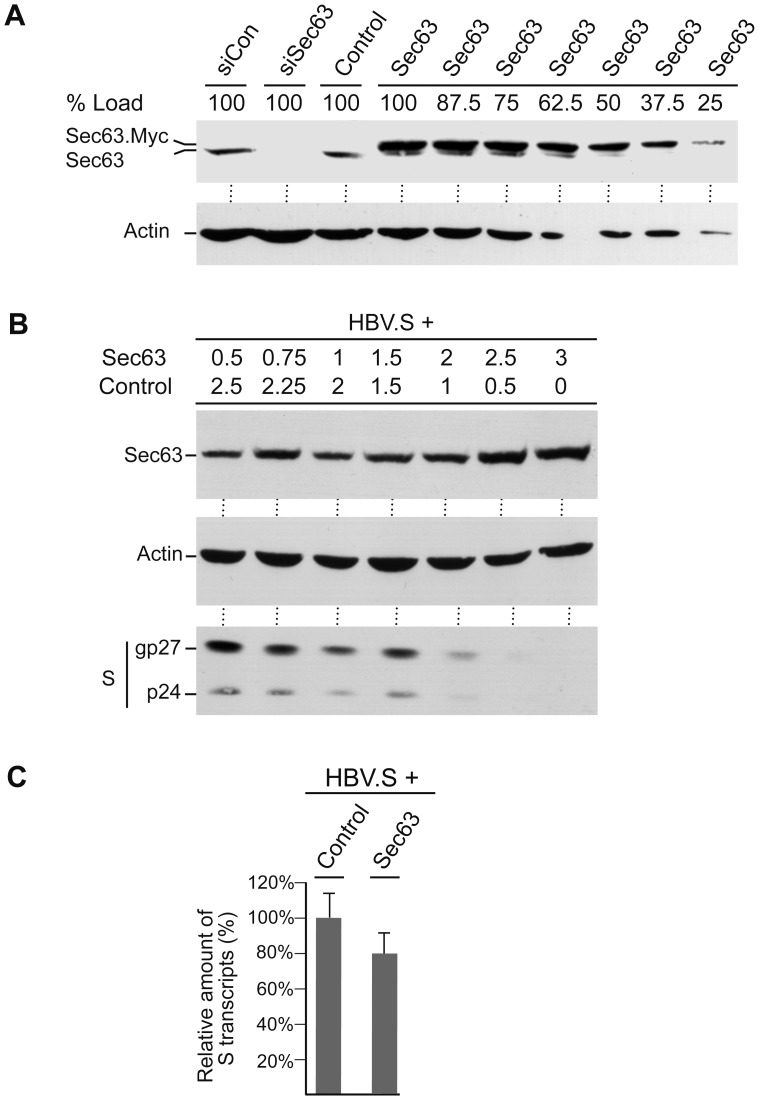
Overexpressing of Sec63 reduces the steady-state HBV.S level in a dose-dependent manner without grossly affecting transcription. **A.** HuH-7 cells were either treated with control siRNA (siCon) or siRNA duplexes targeting Sec63 (siSec63) or were transfected with control DNA (Control) or Myc-tagged Sec63 (Sec63; 3 µg DNA). NP-40 cell lysates were prepared after three days and probed by anti-Sec63 WB. For calculation of the degree of Sec63 overexpression, the Sec63-transfected lysate was serially diluted prior to gel loading as indicated above the lanes (% Load). **B.** Cells were transfected with HBV.S (1 µg DNA) together with increasing amounts of Sec63 DNA, as indicated above the lanes (in µg). Total DNA used for transfections was adjusted with empty plasmid DNA (Control). Extracts were prepared with SDS lysis buffer two days after transfection and assayed by Myc- (top), β-actin- (middle), and HA-specific (bottom) WB. **C.** Cells were cotransfected with HBV.S and control or Sec63 plasmids as in Fig. 1B. HBV.S-specific transcripts were measured by quantitative reverse transcription PCR and are demonstrated in percent amount relative to control cells. Transfections and PCR runs were performed in duplicate, and error bars are standard error of the mean.

To ascertain the degree of Sec63 overexpression, total Sec63 levels were examined in control- versus Sec63-transfected HuH-7 cells by using a polyclonal Sec63-specific antiserum for Western blotting. In order to ensure the true identification of endogeneous Sec63, cells were depleted for Sec63 by treatment with specific siRNA duplexes. As shown in Fig. 2A, endogeneous Sec63 was absent in lysates of siSec63-treated cells. Due to the Myc-tag, exogeneous Sec63 displayed a slower electrophoretic mobility that allowed its distinction from the endogenous isoform (Fig. 2A). Upon serial dilution of the Sec63-transfected lysate and quantification, we calculated that exogeneous Sec63 was overexpressed about 3.2-fold. An accompanying titration experiment showed that Sec63 down-regulated HBV.S in a dose-dependent manner (Fig. 2B). Thereby, the most prominent decline in the HBV.S steady state level appeared when the concentration of the transfected Sec63 DNA was raised above 1.5 µg/10^6^ cells (Fig. 2B). To rule out the possibility that up-regulated Sec63 had effects on mRNA production or stability, the HBV.S-specific transcript levels were measured by quantitative reverse transcription PCR. Although we noticed a modest decline of the HBV.S-specific mRNA in Sec63-overexpressing cells (Fig. 2C), this drop is unlikely to account for the impact of Sec63 on the steady-state HBV.S pool.

### Overexpressed Sec63 Reduces the Steady-state Levels of Polytopic HBV Envelope Proteins Irrespective of Secretion, N-glycosylation and Cell Type

**Figure 3:**
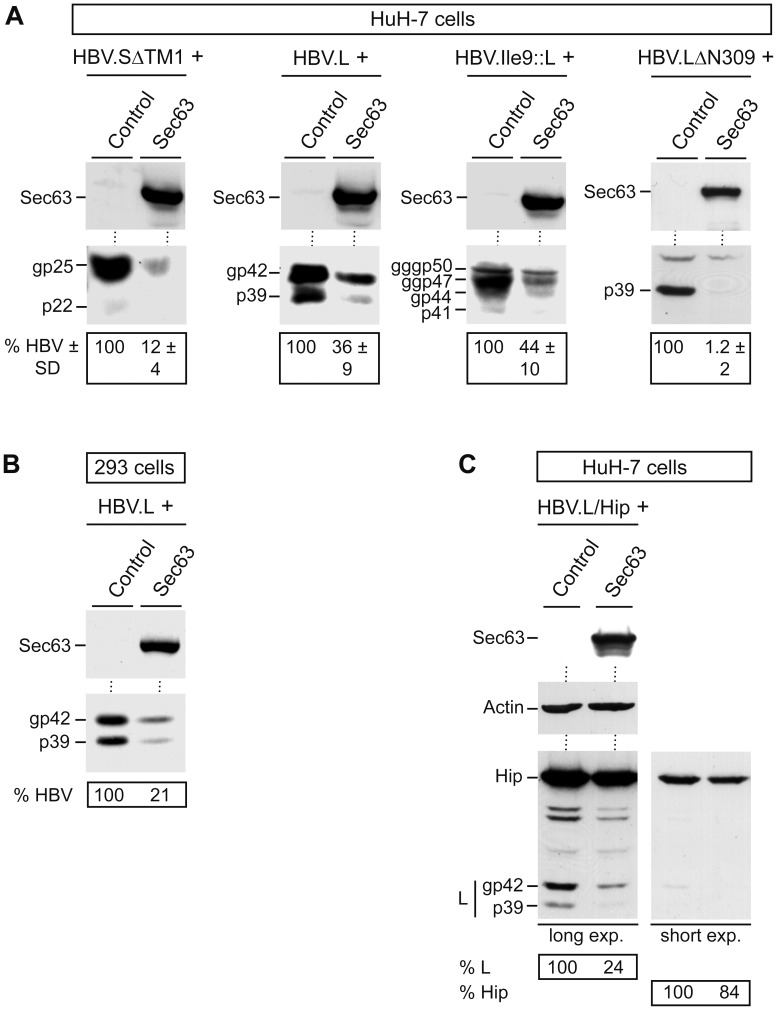
Overexpressing of Sec63 reduces steady-state HBV envelope protein levels irrespective of the secretion status, N-glycosylation, and cell type. **A.** HuH-7 cells were transfected with HA-tagged HBV.SΔTM1, HBV.L, HBV.Ile9::L, or the N-glycosylation-defective HBV.LΔN309 mutant in combination with control DNA or Myc-tagged Sec63 at a 1∶3 ratio, respectively. Cell lysates were probed by anti-Myc (top) and anti-HA (bottom) WB. Non-glycosylated (p) and glycosylated (gp, ggp, gggp) forms of the HBV constructs are indicated on the left of each panel. Cell-associated envelope proteins (HBV) were quantitated and demonstrated in percent amount ± SD relative to control cells (*n* = 3). **B.** HEK293T cells were cotransfected with HA-tagged HBV.L and Myc-tagged Sec63 or control DNA and analyzed by specific WB as outlined in A. **C.** Cells were transfected with the bicistronic plasmid HBV.L/Hip encoding expression units for HA-tagged HBV.L and HA-tagged Hip. Cell extracts were probed by anti-Myc (top), anti-β-actin (middle), and anti-HA (bottom) WB. Long and short exposures of the HA-specific blot plus quantification data are shown.

To specify the role of excess Sec63, we next analyzed the fate of an HBV.S mutant lacking the first TM segment (HBV.SΔTM1). This mutant still enters the ER pathway but fails to assemble into intralumenal secretory SVPs [27]. Upon coexpression with Sec63, the intracellular amount of HBV.SΔTM1 was drastically decreased (Fig. 3A), similar to the situation of wild-type (wt) HBV.S. Thus, excess Sec63 depleted HBV.S proteins irrespective of their secretory phenotypes and therefore likely acts prior to SVP assembly and secretion. To sustain this finding, the outcome of further HBV envelope proteins was studied. The L envelope protein of HBV (HBV.L) is unable to assemble into secretory SVPs due to its dual transmembrane topology [21], [25]. This deficit can be abrogated by the addition of a foreign signal sequence to the N-terminus of HBV.L that blocks the formation of the dual topology and enables secretion [28]. The overexpression of Sec63 decreased the non-glycosylated and glycosylated forms of both the wt HBV.L and HBV.Ile9::L signal fusion proteins (Fig. 3A). This indicates that excess Sec63 compromises the production of HBV envelope proteins independently of their particular secretory behavior. During ER translocation, the HBV envelope proteins are modified by N-linked glycosylation conducted by the translocon-associated OST complex [5], [29]. Because the overexpression of Sec63 could impair the structural integrity of the OST complex concomitant with translocational constraints, we studied a glycosylation-defective HBV.L mutant (HBV.LΔN309). Due to the mutation of the N-glycan acceptor site, this mutant appeared only in the non-glycosylated p39 form (Fig. 3A). HBV.LΔN309 was also down-regulated by excess Sec63, thus largely excluding a participation of the N-linked glycosylation machinery in this process.

Since HBV is a hepatotropic virus, we routinely used HuH-7 cells for viral expression studies. Some liver cells, however, have been implicated in Sec63 abnormalities, as mutations in the Sec63 gene contribute to the development of autosomal dominant polycystic liver disease [1], [17]. Therefore, we examined the effect of excess Sec63 in HEK293T human embryonic kidney cells in parallel. As exemplified for the HBV.L protein, overexpressed Sec63 reduced the intracellular HBV.L level to almost the same extent as compared to HuH-7 cells (Fig. 3B), arguing against cell type-specific effects. In combining these data, we deduced that excess Sec63 diminished the steady-state pools of the HBV envelope proteins synthesized at the ER.

However, there remained the possibility that the observed effects were simply due to an exceptionally high degree of cotransfection of the Sec63 and HBV constructs, thereby provoking intracellular imbalances, unspecific protein interferences, and/or other effects. To address this issue, we used a bicistronic plasmid vector carrying expression units for HA-tagged HBV.L under the control of the human metallothionein IIa (hMTIIa) promoter and for HA-tagged Hip (Heat shock cognate protein 70 [Hsc70] interacting protein) under the control of the cytomegalovirus (CMV) promoter [22]. Human Hip is a cytosolic cochaperone involved in the regulation of Hsc70 chaperone activity. This construct offered the chance to monitor the effect of excess Sec63 in the same cells and the same blot. Upon cotransfection of HBV.L/Hip with Sec63, the level of HBV.L was again reduced, while the amount of cytosolic full-length Hip was only marginally affected (Fig. 3C). For unknown reasons, we noted, however, that Hip-specific degradation products were sensitive to excess Sec63.

### Overexpressed Sec63 acts during HBV.S Biogenesis in a Proteasome-independent Manner

**Figure 4:**
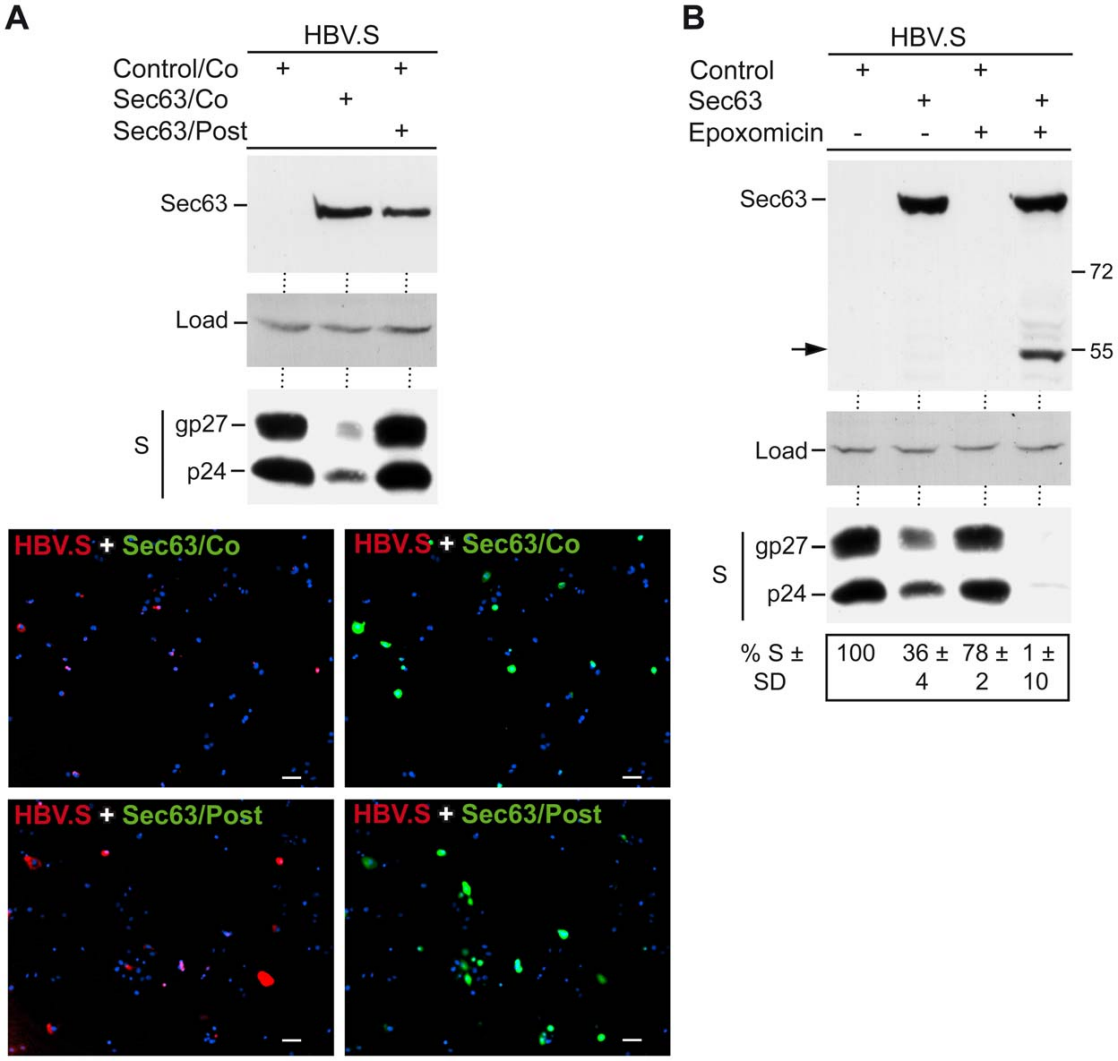
Overexpressing of Sec63 acts during HBV.S formation in a proteasome-independent manner. **A.** HBV.S was transfected with control DNA or Myc-tagged Sec63 at a 1∶3 ratio, respectively, either simultaneously (Control/Co and Sec63/Co) or postponed (Sec63/Post). In the latter case, cells transfected with HBV.S were retransfected with Sec63 two days later and harvested after additional 24 h. Cell lysates were probed by anti-Myc (top) and anti-HA (middle bottom) WB. Uniformity of sample loading is shown by a band cross-reacting with the HA antibody (load). Immunofluorescence analysis of HBV.S+Sec63/Co- and HBV.S+Sec63/Post-transfected cells. Representative pictures are shown in the bottom. Cells were stained with mouse anti-Myc and rat anti-HA antibodies followed by staining with AlexaFluor 488-conjugated anti-mouse and AlexaFluor 546-conjugated anti-rat antibodies. DNA staining of the nuclei is in blue. Bar, 50 µm. **B.** For proteasomal inhibition, cells were cotransfected with HBV.S plus control DNA or Myc-tagged Sec63 and were mock-treated or treated with epoxomicin for 16 h. Cell lysates were analyzed as in A. The arrow to the left of the top panel depicts degradation products of Sec63. HBV.S levels are demonstrated in percent amount ± SD relative to control cells (*n* = 3).

To get insights into the underlying mechanism(s), we investigated the time frame of the action of up-regulated Sec63. Although the data presented so far favor a cotranslational mode of Sec63 activity, posttranslational impacts of excess Sec63, such as a disruption of the integrity of the ER membrane, could not be ruled out. Therefore, cells transiently expressing the HBV.S construct for two days were retransfected with Sec63 and analyzed after an additional day. For comparison, cells were simultaneously cotransfected with HBV.S and Sec63. As shown in Fig. 4A, overexpressed Sec63 did not affect a preexisting HBV.S pool. To exclude the possibility that the lack of Sec63 action in the “posttransfection” experiment was due to constraints in the retransfection, we analyzed the rate of cotransfection by immunofluorescence. Although the intensity of the HBV.S-specific signals was substantially lower as compared to cells expressing HBV.S alone, the amplification of the signals enabled the detection of transfected cells. Upon quantification, 49±14% or 42±7% of transfected cells were determined to be co- or posttransfected with Sec63, respectively (*n* = 5) (Fig. 4A). Together, these data support a role for Sec63 at an early stage of HBV.S protein synthesis, possibly occurring during translocation.

In yeast, Sec63p and its Kar2p partner protein have been indicated to be involved in the export of misfolded proteins to the cytosol during ER-associated degradation (ERAD) by the proteasome [30], [31]. For the mammalian Sec63, such a role is ill-defined. To examine whether the up-regulation of Sec63 reduced the HBV.S level via an enforced export out of the ER, transfected cells were treated with the proteasome inhibitor epoxomicin. The efficacy of the drug during the applied assay conditions was indicated by the appearance of C-terminally degraded Sec63 derivatives that accumulated in epoxomicin- but not in mock-treated cells (Fig. 4B). Importantly, the inhibition of the ERAD pathway did not abolish the ability of excess Sec63 to down-regulate HBV.S (Fig. 4B), suggesting that the reduction of the HBV.S steady-state level is not mediated by proteasomal degradation. To account for this unexpected finding, different explanations are conceivable. First, non-proteasomal proteases, like amino- and carboxypeptidases, may contribute to the destruction of improperly translocated HBV.S chains. Such proteases, acting in the cytosol and the ER/secretory pathway, have been reported to participate in endogenous antigen processing [32]. Second, excess Sec63 may halt HBV.S translocation, thereby promoting cotranslational destruction prior to completion of protein synthesis. In doing so, full-length, C-terminally tagged HBV.S chains would not be detectable. And third, autophagosomes/lysosomes may be involved in the disposal of HBV.S. This pathway has been described in cells permanently replicating HBV [33].

### Overexpressed Sec63 is a Potent Reducer of Steady-state Levels of Polytopic Proteins

**Figure 5:**
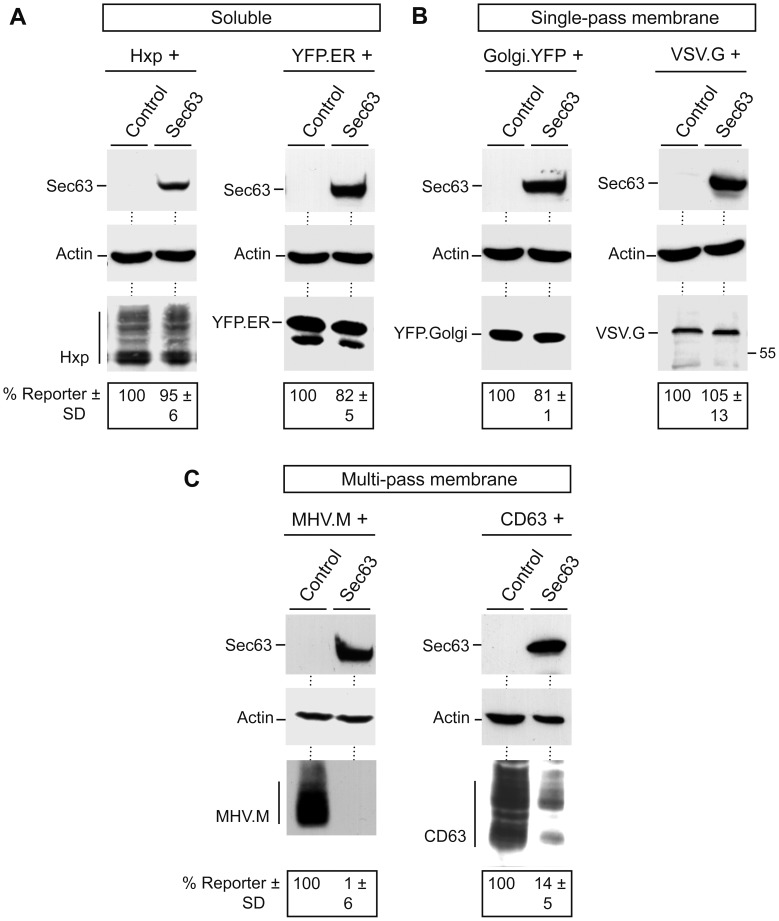
Overexpressing of Sec63 reduces steady-state levels of polytopic proteins. ER reporter constructs, including soluble (**A**), single-pass membrane (**B**), and multi-pass membrane (**C**) proteins were used for cotransfection studies as indicated above the panels. Each reporter was transfected with control DNA or Myc-tagged Sec63 at a 1∶3 DNA ratio, respectively, into HuH-7 cells. Three days later, cell lysates were subjected to specific immunoblotting. Ectopic expression of Sec63 was assessed with anti-Myc antibodies, while anti-β-actin antibodies were used to demonstrate identical gel loading. Hxp, MHV.M, and CD63 were probed with anti-HA antibodies, YFP.ER and Golgi.YFP were detected with anti-GFP antibodies, and VSV.G was verified with anti-G-specific antibodies. Reporter expression values were determined by densitometry and demonstrated in percent amount ± SD relative to control cells (*n* = 3).

Although being clients of Sec63, the HBV envelope proteins do not directly interact with Sec63 as measured in a split-ubiquitin yeast two-hybrid assay (data not shown). This prompted us to examine whether or not Sec63 may play a more general role in the production of ER proteins. To this aim, we employed a panel of different translocational reporter proteins. For soluble ER marker proteins, hemopexin (Hxp) and a yellow fluorescent ER fusion protein (YFP.ER) were used that are cotranslationally translocated across the ER membrane by virtue of their N-terminal cleaved signal peptides [34]. While YFP.ER is retained within the ER lumen via its C-terminal KDEL retention signal, Hxp is secreted from the cells. As shown in Fig. 5A, both proteins were efficiently synthesized in transfected HuH-7 cells. Because Hxp is a substrate to N- and O-linked glycosylation [34], it appeared in multiple forms with different electrophoretic mobilities. Upon overexpression of Sec63, the intracellular levels of both reporters as well as the glycosylation pattern of Hxp were largely unaffected, indicating that excess Sec63 did neither affect ER translocation nor the steady-state levels of these soluble proteins. Identical results were obtained when ER membrane proteins with a single membrane-spanning segment were analyzed. Here, we took use of either a Golgi.YFP fusion construct encoding YFP with a N-terminal membrane anchoring signal peptide of β1,4-galactosyltransferase or the G glycoprotein of vesicular stomatitis virus (VSV.G). VSV.G harbors a cleavable signal peptide and a single TM segment in its C-terminus and is a substrate to N-linked glycosylation [35], [36]. Coexpression studies demonstrated that Sec63 neither down-regulated Golgi.YFP nor VSV.G (Fig. 5B). It also did not affect the glycosylation pattern of VSV.G. Since these data are in seemingly conflict with the results obtained for the HBV envelope proteins, we next assessed the behavior of multi-spanning membrane proteins synthesized in the presence of up-regulated Sec63. The M envelope protein of the mouse hepatitis coronavirus (MHV.M) is a triple-spanning protein with its first and second TM segments serving as a signal-anchor and stop-transfer signal, respectively [37]. Beside viral proteins, human CD63/LAMP-3 (lysosomal-associated membrane protein 3), a member of the tetraspanin family, was included as a cellular multi-pass reporter [38]. HA-tagged versions of MHV.M and CD63 were cotransfected with Sec63 and cell extracts were probed by Western blotting. Consistent with published data [37], [38], the MHV.M and CD63 proteins run as broad smear in SDS-polyacrylamide gels likely due to glycosylation (Fig. 5C). Importantly, overexpressed Sec63 almost completely depleted each of the multi-pass reporters (Fig. 5C), as it is the case for the HBV envelope proteins.

**Figure 6:**
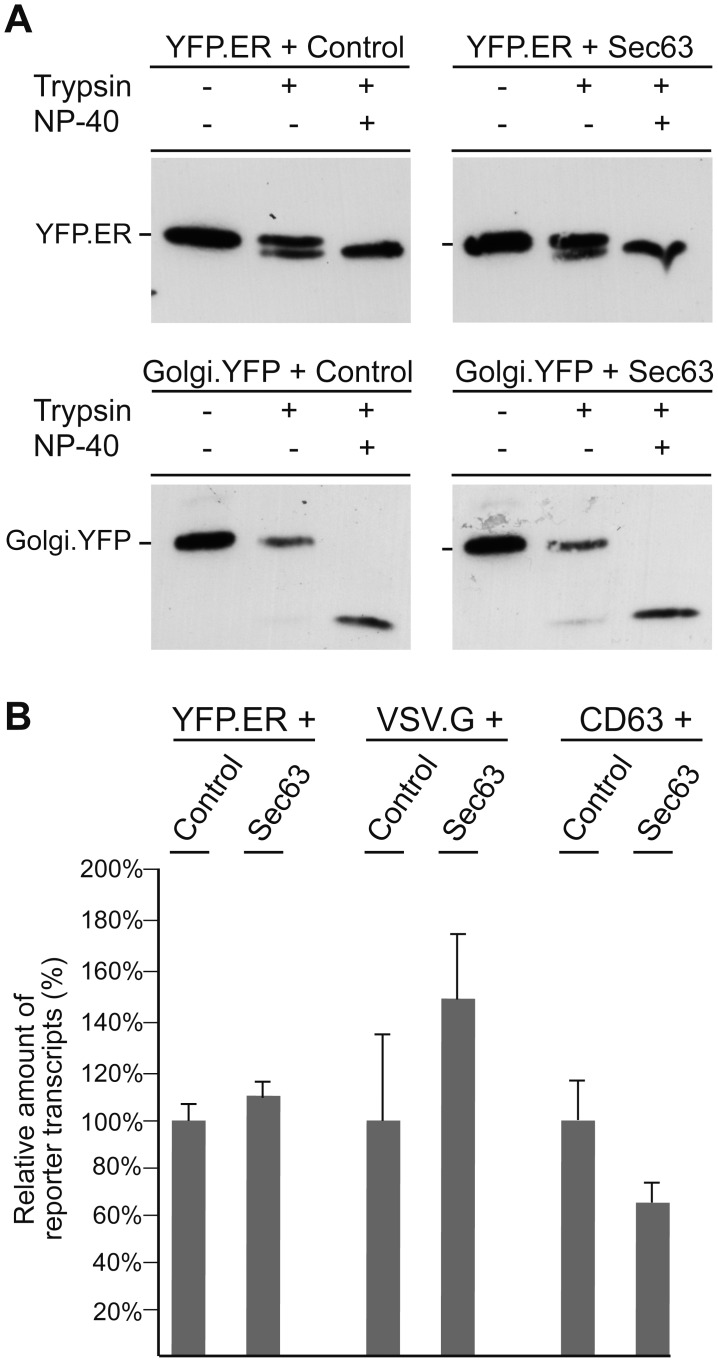
Overexpressing of Sec63 neither inhibits ER import of ER.YFP and Golgi.YFP nor grossly affects reporter gene transcription. **A.** Cells were transfected with ER.YFP or Golgi.YFP together with control DNA or Myc-tagged Sec63, exactly as in Fig. 5. Microsomes were prepared and either left untreated or digested with trypsin in the absence or presence of NP-40, as indicated above the lanes. Samples were analyzed by GFP-specific WB. **B.** Cells were cotransfected with the indicated reporter constructs plus control or Sec63 plasmids as in Fig. 5. Reporter-specific transcripts were measured by quantitative reverse transcription PCR and are demonstrated in percent amount relative to control cells. Transfections and PCR runs were performed in duplicate, and error bars are standard error of the mean.

To probe whether the non-glycosylated YFP.ER and Golgi.YFP reporters were truly translocated into the ER of Sec63-overexpressing cells, trypsin protection experiments were performed. Microsomes of cotransfected cells were prepared and either left untreated or treated with trypsin in the absence or presence of detergent. For both reporters, protection against trypsin was observed in intact microsomes (Fig. 6A). On disruption of microsomes with detergent, trypsin completely converted the proteins to tryptic fragments thereby leading to the disappearance of full-length YFP.ER and Golgi.YFP. We noted, however, that neither construct was fully protected from proteolysis in the absence of detergent (Fig. 6A), likely as a consequence of some leakage of the microsomes. Nonetheless, the amounts of protected YFP.ER and Golgi.YFP chains were almost identical in control- and Sec63-overexpressing cells. Thus, up-regulated Sec63 does not interfere with the translocation of these substrates.

As measured by quantitative reverse transcription PCR of representative reporter genes, up-regulated Sec63 does also not impair the synthesis and stability of YFP.ER- and VSV.G-specific mRNAs (Fig. 6B). In the case of CD63, transcript levels were diminished in the presence of exogenous Sec63 (Fig. 6B). This may in part, but not entirely contribute to the observed decline of the CD63 protein level (see Fig. 5C).

### Overexpressed Sec63 Affects Cotranslational Membrane Protein Insertion rather than Translation

**Figure 7:**
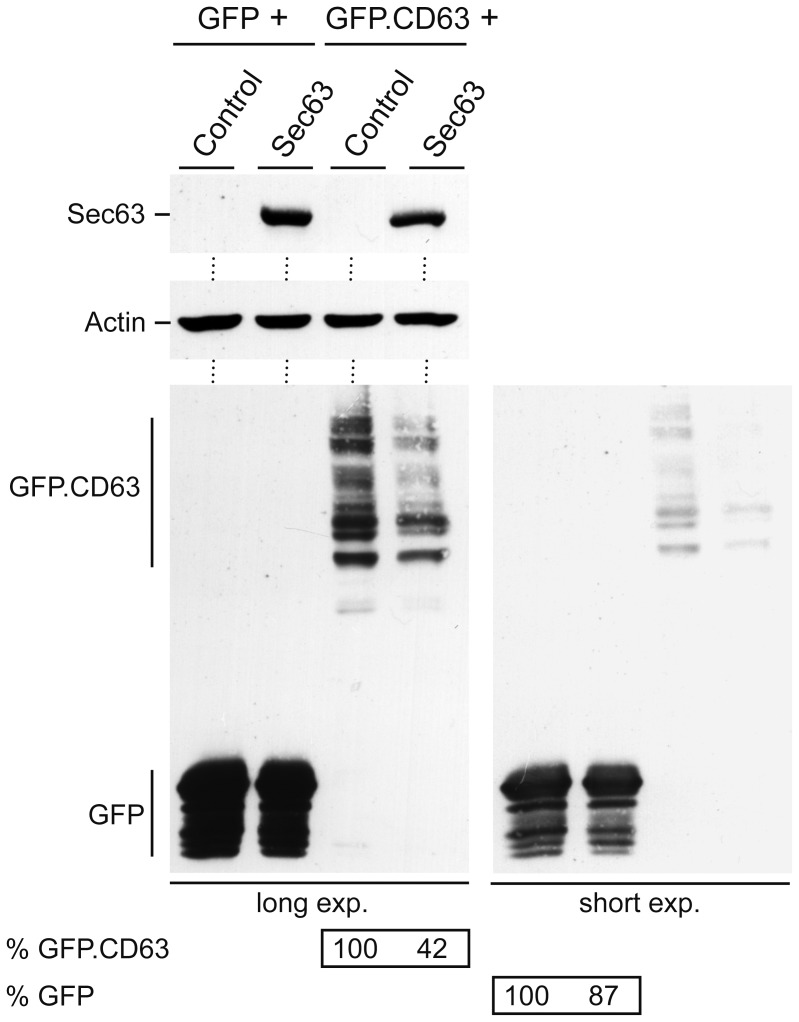
Overexpressing of Sec63 reduces the cotranslational insertion of GFP.CD63 without grossly affecting the translation of GFP. HuH-7 cells were transfected with GFP or GFP.CD63 together with control DNA or Myc-tagged Sec63, as in Fig. 5. Cell extracts were prepared by a 15 minutes boiling in SDS sample buffer and probed by anti-Myc (top), anti-β-actin (middle), and anti-GFP (bottom) WB. Long and short exposures of the GFP-specific blot plus quantification data are shown.

In order to determine the step(s) at which Sec63 is acting, we analyzed whether up-regulated Sec63 affected the translation or cotranslational insertion of the multi-pass CD63 reporter. For this, we used two constructs encoding the cytosolic green fluorescent protein (GFP) or GFP with a C-terminal fusion of CD63 (GFP.CD63). Both constructs were in identical vector backbones and under the control of identical transcriptional elements. Upon cotransfection of either of the two constructs with control or Sec63 DNA, their common GFP-tag enabled immunodetection on the same gel. Like the CD63 wt protein (see Fig. 5C), the GFP.CD63 fusion protein appeared in multiple bands likely as a result of glycosylation, implicating that it had entered the ER/secretory pathway (Fig. 7). Up-regulated Sec63 diminished the yield of the GFP.CD63 membrane reporter, while it had little effects on the cytosolic GFP protein (Fig. 7). Since the transcripts of the two reporters were generated from the same promoter/polyadenylation elements and hence should be similar, we inferred that excess Sec63 appeared to affect the cotranslational insertion rather than the translation of polytopic proteins.

### Down-regulated Sec63 is a Potent Amplifier of the Steady-state Levels of Polytopic Proteins

**Figure 8:**
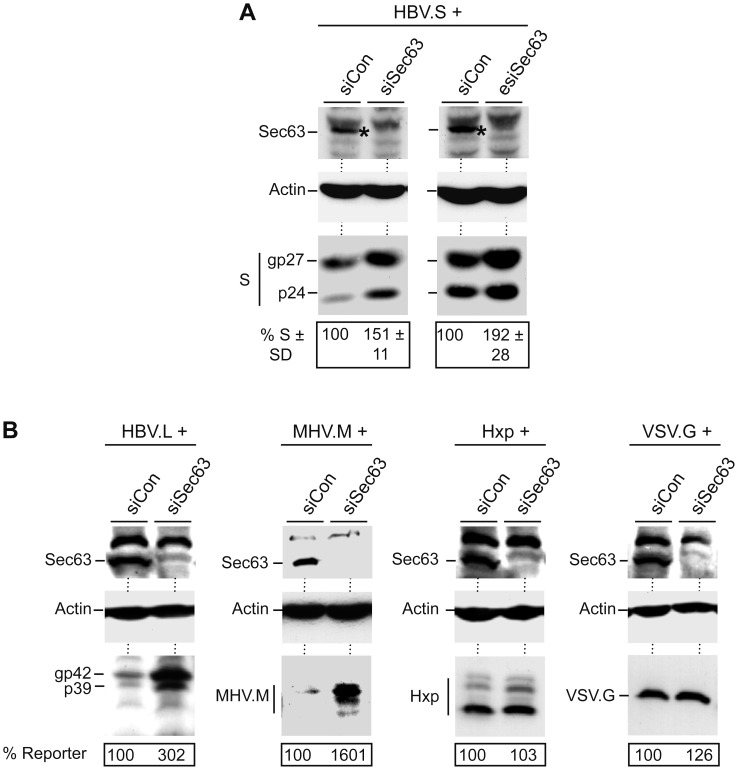
Silencing of Sec63 increases the steady-state levels of polytopic proteins. **A.** HuH-7 cells were treated with control siRNA (siCon) or siRNA duplexes (siSec63) and esiRNA molecules (esiSec63) targeting Sec63. Two days later, cells were transfected with HBV.S and lysed after 48 h. Lysates were subjected to Sec63-specific WB to examine depletion. For HBV.S detection, anti-HA WB was used. To confirm equal loading of lysates, β-actin levels were assessed. Below the panels, the cell-associated HBV.S levels are demonstrated in percent amount ± SD relative to control cells (*n* = 2). **B.** Cells were transfected with control or Sec63-specific siRNAs as above, prior to transfection with the indicated ER reporters. Hxp was used as a soluble marker, VSV.G represented a single membrane-pass protein, while HBV.L and MHV.M served as multi-spanning membrane reporters. Cell lysates were subjected to immunoblotting using anti-Sec63 (top), anti-β-actin (middle), or anti-HA/VSV.G antibodies (bottom). Reporter expression values were determined by densitometry and demonstrated in percent amount relative to control cells.

Collectively, these data suggest that excess Sec63 interferes selectively with the formation of multi-spanning ER proteins. Therefore, we next analyzed ER cargoes in Sec63-deficient cells. For depletion of Sec63, classic siRNA duplexes (siSec63) as well as siRNA molecules prepared by endoribonuclease digestion (esiSec63) were transfected into HuH-7 cells prior to transfection with the HBV.S construct. Western blot analysis of cell lysates showed that both siRNA species depleted Sec63 by about 80% as compared to control siRNA-treated cells (Fig. 8A). When lysates were probed for HBV.S, the knock-down of Sec63 resulted in significant higher intracellular p24/gp27 levels as compared to control-treated cells. Of note, the intracellular increase of HBV.S was not due to a block in SVP secretion, since the extracellular amount of HBV.S was also elevated in Sec63-depleted cells (data not shown). These results indicate that Sec63 is *per se* not required for HBV.S production; rather, its limitation promotes HBV.S synthesis and/or stability. To corroborate these results, other ER reporter proteins were studied under conditions of deficit Sec63. As representative markers, the soluble Hxp protein, the single membrane-spanning VSV.G protein, and the multiple-spanning proteins HBV.L and MHV.M were investigated. Depletion efficacy of Sec63 and intracellular steady-state levels of the marker proteins were monitored by specific immunoblotting. As shown in Fig. 8B, the biogenesis of Hxp and VSV.G were unaffected by the knock-down of Sec63. Conversely, the amounts of each of the multi-spanning reporters were increased in Sec63-depleted cells. These observations suggest that human Sec63 is not essential for cotranslational ER translocation, but regulates the translocation efficiency of certain precursor polypeptides.

### Overexpressed ERdj1 and ERdj4 do not Affect the Steady-state Pool of the Polytopic HBV.S Envelope Protein

**Figure 9:**
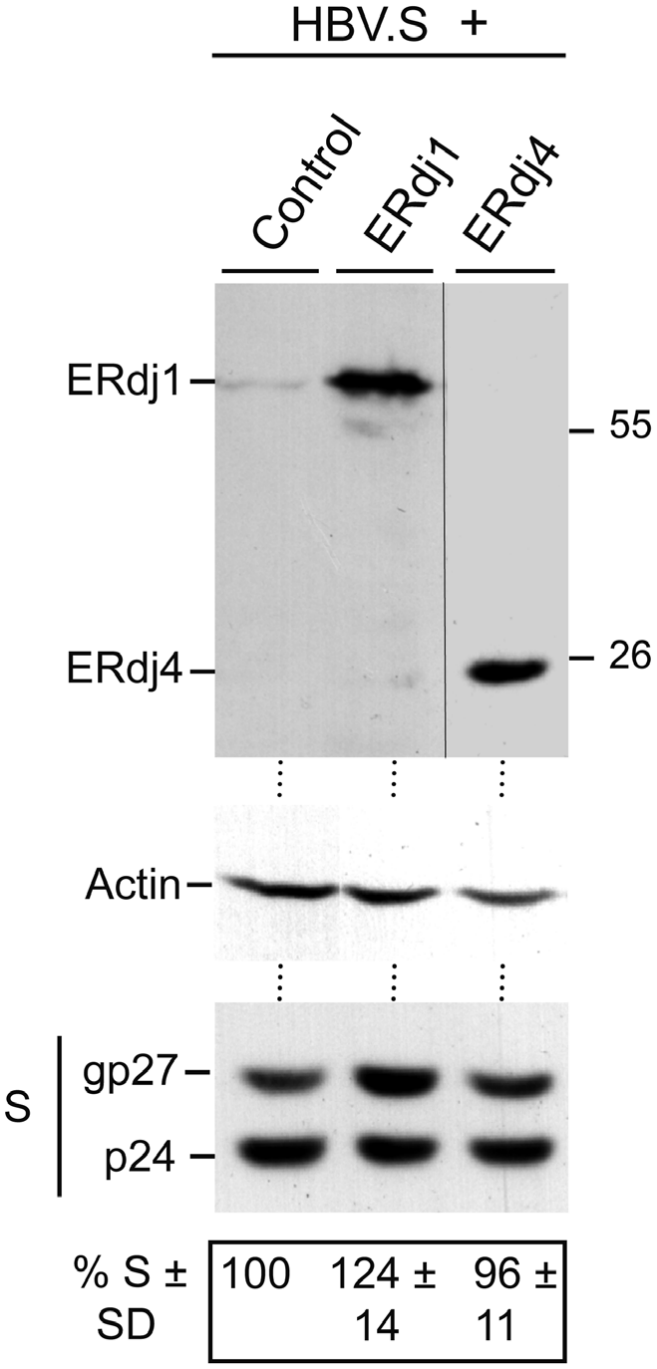
Overexpressing of ERdj1 and ERdj4 does not affect the steady-state HBV.S level. HBV.S was cotransfected with control DNA or FLAG-tagged ERdj1 and ERdj4 at a 1∶3 DNA ratio, respectively, into HuH-7 cells. After transient expression for three days, lysates were prepared and assayed by WB using anti-FLAG (top), anti-β-actin (middle), or anti-HA antibodies (bottom). Relative HBV.S expression values ± SD (*n* = 3) were determined as in Fig. 1.

Beside Sec63 (*i.e.*, ERdj2), six other resident ER proteins with J domains inside the ER lumen have been described in mammals [11]. ERdj1 is a further translocon-associated ERdj protein and constitutes a single-pass type I membrane protein. Unlike Sec63, it has the ability to associate with translating ribosomes, likely to modulate translation [9]. ERdj4 is a soluble ER protein and plays a role in the degradation of ERAD substrates, but has not been found in close contact with the Sec61 translocon [39]. To analyze whether an up-regulation of these ERdj proteins might also affect the level of a multi-spanning membrane protein, FLAG-tagged versions of ERdj1 and ERdj4 were cotransfected with HBV.S. FLAG-specific Western blotting confirmed the ectopic expression of ERdj1 and ERdj4 in 63 and 25 kDa forms, respectively, consistent with their theoretical molecular masses (Fig. 9). Importantly, when lysates were probed for HBV.S, no significant change in the p24/gp27 level was noticeable (Fig. 9). Thus, in contrast to Sec63, the overexpression of neither ERdj1 nor ERdj4 compromises the production and/or life span of HBV.S.

### The Role of BiP in the Action of Overexpressed Sec63

**Figure 10:**
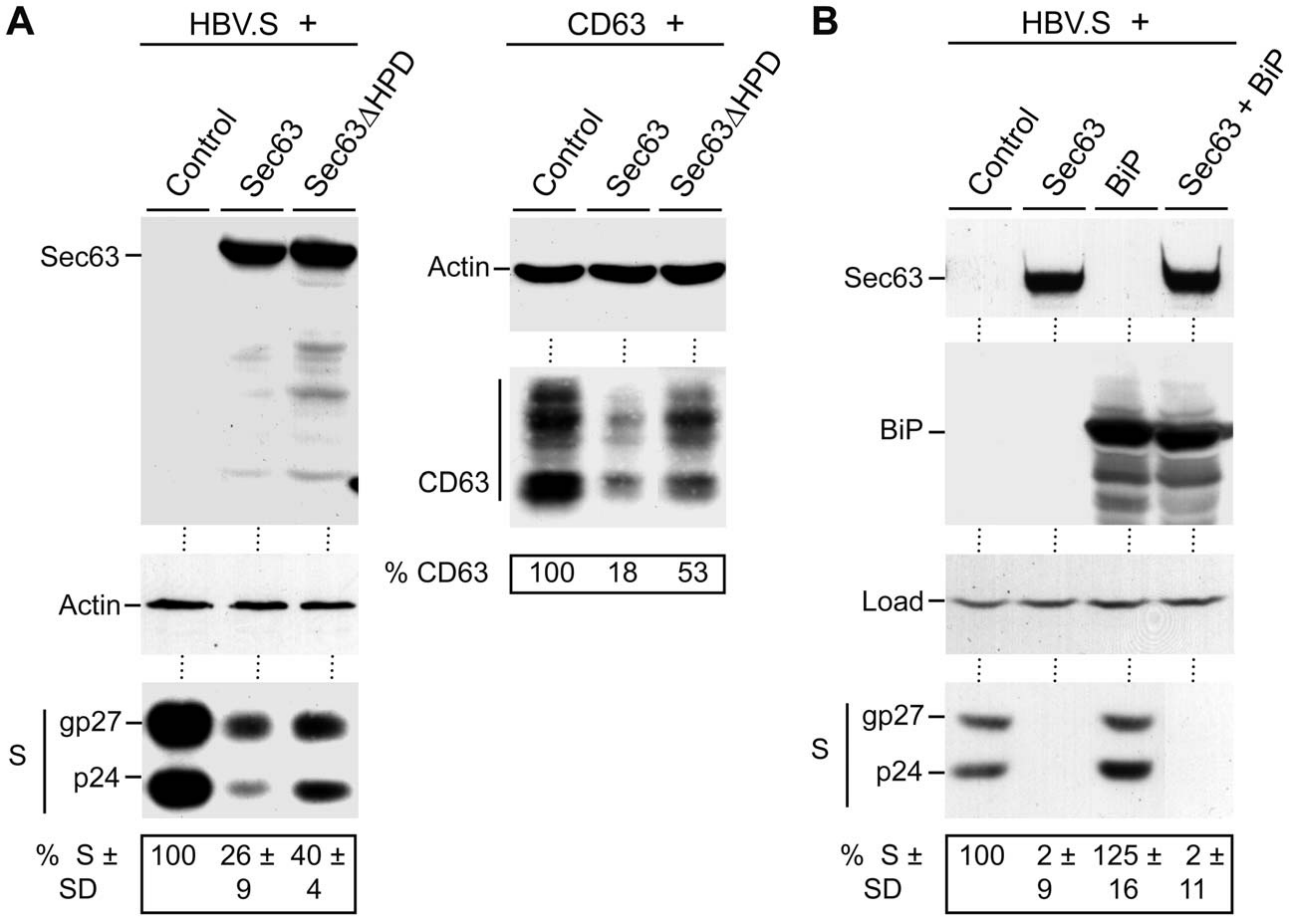
Role of BiP in the action of overexpressed Sec63. **A.** HuH-7 cells were cotransfected with HBV.S (left panels) or CD63 (right panels) in combination with control DNA or the Myc-tagged wt Sec63 or mutant Sec63ΔHPD at a 1∶3 DNA ratio, respectively. In case of HBV.S, cellular lysates were subjected to anti-Myc (top), anti- β-actin (middle), and anti-HA (bottom) WB. Relative HBV.S expression values ± SD (*n* = 4) were determined as in Fig. 1. In case of CD63, lysates were probed by anti-β-actin (top) and anti-HA (bottom) WB. **B.** For cotransfections with HBV.S, control DNA, Myc-tagged Sec63, and FLAG-tagged BiP were used as a 1∶3 or 1∶3∶3 DNA ratio, respectively. Cell extracts were probed by WB with antibodies against Myc (top), FLAG (top middle), or HA (bottom). Uniformity of sample loading is shown by a band cross-reacting with the HA antibody (load; bottom middle). Relative HBV.S expression values ± SD (*n* = 2) were determined as in Fig. 1.

In the next set of experiments, we focused on the Sec63/BiP interplay. The yeast and mammalian Sec63 proteins have been shown to interact with the ER lumenal Kar2p/BiP chaperone in a productive manner [10], [12], [15]. These interactions require their lumenally exposed J domains containing the highly conserved His-Pro-Asp (HPD) motif [11], [40], [41]. Because mutations of this sequence abolish the functional interaction between J domains and their chaperone partners [40], [41], we reasoned to study a Sec63 mutant devoid of the HPD motif (Sec63ΔHPD). The wt and mutant Sec63 proteins were coexpressed with HBV.S and cell extracts were examined by specific immunoblotting. The Sec63ΔHPD mutant likewise reduced HBV.S, albeit its down-regulating activity was reproducibly less pronounced as compared to wt Sec63 (Fig. 10A). Similar results were obtained with the polytopic CD63 reporter (Fig. 10A). These results suggest that the stimulation of the ATPase activity of BiP by the J domain of Sec63 may not be mandatory for the role of Sec63 in polytopic membrane homeostasis. However, they do not thoroughly exclude that Sec63 may function in concert with BiP. To dissect whether Sec63 *per se* or the associated BiP chaperone is responsible for HBV.S protein reduction, the reporter was studied in cells overexpressing Sec63 and BiP either individually or together. The up-regulation of BiP neither reduced the level of HBV.S nor reverted the inhibitory action of excess Sec63 on HBV.S (Fig. 10B). Together, these data suggest that Sec63 is primarily responsible for the control of membrane protein levels, possibly with aid of BiP.

## Discussion

This work provides support for a role of human Sec63 in protein biogenesis at the ER. By analyzing the effects of excess and deficit Sec63 in human cell lines, we observed an inverse correlation between the cell content of Sec63 and the level of particular ER substrates. As reporters, we used soluble, single- and multi-spanning membrane proteins of viral, bacterial, and mammalian origin and observed that the overexpression of Sec63 specifically reduces the steady-state levels of polytopic membrane proteins, while the limitation of Sec63 has reciprocal effects. The data of the kinetic experiment support a role for Sec63 at an early stage of multi-spanning membrane protein synthesis, and we favor a model where this occurs during translocation. Overall, our data implicate that Sec63 has a regulatory function during cotranslational insertion of multi-pass membrane proteins that is, however, not essential. Its action as a translocational controller may explain why the loss of Sec63 function associated with polycystic liver disease (PCLD) is not lethal in humans [1], [17]. With regard to our finding that the depletion of Sec63 augments the biogenesis of polytopic proteins, dysfunctional Sec63 may evoke an unbalanced physiological situation in PCLD patients, thereby eventually affecting proteins that are involved in the control of biliary cell growth and proliferation.

Previous studies have shown that the yeast and mammalian Sec62 and Sec63 proteins interact with each other [13], [14], [42], suggesting a concerted action of these proteins. While this is true for posttranslational protein translocation into the yeast ER [2], [39], overlapping roles for the mammalian proteins have not been documented. Rather, *in vitro* biochemical analyses of dog and bovine pancreatic microsomes revealed that only small fractions of Sec62 and Sec63 are associated with each other, while the majority of these proteins were not found in a complex [10], [13]. Cross-linking experiments in cell-free systems also argue for individual functions of mammalian Sec62 and Sec63 proteins, as Sec63 was found in transient proximity to newly synthesized membrane proteins at early stages of membrane integration, while Sec62 was detected at a later stage [18]. Consistent with this, we show that the overexpression of Sec62 did not limit HBV.S membrane protein expression. Moreover, up-regulated Sec63 exerts its inhibitory activity independent of the Sec62 protein content of the cells and independent of its Sec62-interacting motif, indicating that a stoichiometric complex formation between Sec63 and Sec62 may not be mandatory for function. A Sec62-independent action of Sec63 is also used by the parasite *Trypanosoma brucei* that lacks a Sec62 ortholog, but engages Sec63 for co- and posttranslational ER protein import [43].

The substrate-specificity of the Sec63 action is intriguing, as it selectively targets multi-spanning membrane proteins. A currently unresolved issue in membrane protein folding is how and when TMs are released from Sec61 once translocation is terminated. One view, supported by lipid cross-linking studies is that TMs are dislocated from the translocon and move into the bilayer by passive thermodynamic partitioning through a lateral cleft in the Sec61α subunit. Hence, the driving force for the lateral exit of TMs can be correlated to their intrinsic hydrophobicity. Numerous studies have shown that such a model is likely to apply for the membrane integration of single-spanning membrane proteins [44], [45], [46]. The biogenesis of polytopic membrane proteins, however, presents distinct challenges to the ER translocon and demands additional modulation. Experimental data suggested that up to four TMs of one polypeptide could be present in the translocation channel at the same time concomitant with widening of the pore [47], [48], [49]. Moreover, TMs appear to progress through different proteinaceous environments prior to their integration into the lipid bilayer either one by one, as pairs or even groups, suggesting the participation of additional factors beside the translocon core complex [50], [51], [52]. Because mammalian Sec63 is adjacent to Sec61 [10] and because it selectively influences the synthesis of polytopic membrane proteins, it is tempting to speculate that Sec63 may affect the structure and functionality of those translocons handling polytopic proteins. In this view, Sec63 (i) may control expansion of the translocon to accommodate multiple TMs, (ii) may act as a lateral gate keeper controlling the opening and closure of the exit portal towards the lipid layer, and/or (iii) may function as a chaperone or an escort for TMs awaiting lipid integration. A regulatory function of Sec63 may also explain why it is dispensable for cotranslational translocation into reconstituted proteoliposomes [6].

Since excess Sec63 inhibits the biogenesis of polytopic proteins, while its deficit has inverse effects, Sec63 has features of a “negative” regulator. The concept of translocational regulation by substrate-selective *trans*-acting factors has been well documented [53]. For example, the translocon-associated TRAM and TRAP proteins can interact directly with some nascent chains and stimulate translocation in a signal sequence–dependent manner, but neither protein is absolutely required because at least some substrates can be translocated in their absence [7], [8], [54]. Translocon-associated ER lumenal factors, like the soluble BiP chaperone or the membrane-embedded OST complex, appear to influence translocation of nascent chains by mechanisms including trapping, modification, and/or folding, thereby facilitating quality control of ER substrate entry [5], [16]. For the translocon-associated Sec63 protein, we suggest a quantity control function in such that it may regulate the amount of polytopic protein integration in order to prevent ER membrane overload.

At first glance, our RNA interference data are in conflict with two recent works, studying the loss of Sec63 function *in vitro* and *in vivo*. By using a mutant mouse model for PCLD, Fedeles *et al.* [55] demonstrated that Sec63 differently affected the polycystic kidney disease gene products, polycystin-1 and polycystin-2. Both proteins are synthesized as multi-spanning proteins at the ER whereupon polycystin-1 uses a cleavable signal peptide, while polycystin-2 employs a non-cleavable signal [56], [57]. In Sec63 knock-out cells, the biogenesis of polycystin-1 was strongly impaired, while polycystin-2 was only moderately affected [55]. By studying seven different lumenal, single- and multi-spanning cotranslational ER reporters with and without cleavable signal peptides, Lang *et al.* [58] showed that some proteins need the help of Sec63, while others do not. The authors suggest that the Sec63-dependancy may be in part related to the properties of cleavable and non-cleavable signal peptides [58]. Except for the HBV.Ile9::L reporter that contains an artificial, presumably uncleaved signal peptide (R. Prange, unpublished observation), all polytopic cargos used in this work contain non-cleavable signal peptides. Hence, apart from its quantity control function Sec63 appears to play an additional “active” role in cotranslational protein import depending on the character of the signal peptide. A greater panel of proteins will have to be analyzed to deduce the exact function of Sec63.

The J domains of yeast and mammalian Sec63 proteins have been demonstrated to functionally interact with the Kar2p/BiP chaperone which plays a role in many functions of the ER, including lumenal gating of the translocon, assisting protein folding, and targeting misfolded proteins for proteasomal degradation [16]. Thus, the up-regulation of Sec63 could lead to trapping of BiP concomitant with defects in ER translocation and homeostasis. However, we do not favor this interpretation, because the overexpression of two other BiP-interacting ERdjs, ERdj1 and ERdj4, did not impair the HBV.S steady-state protein level. Also, the simultaneous overexpression of Sec63 and BiP did not revert the inhibitory effect of excess Sec63 on the HBV.S cargo. Nonetheless, the question remains whether Sec63 acts in conjunction with BiP. In case of the yeast Sec63p, mutations of the hallmark HPD motif of its J domain abolish the functional interaction with Kar2p, *i.e.*, lead to a failure to stimulate the ATPase activity of the chaperone [15], [40]. The overexpression of a corresponding mutant of the human Sec63 compromised the steady-state levels of polytopic reporters but half the amount as compared to the wt protein. One interpretation of this finding may be that BiP is not absolutely required for the Sec63 action. Alternatively, the HPD mutation may weaken but may not completely prevent the Sec63/BiP interaction. In favor of the latter interpretation are observations that J domain proteins can form stable complexes with Hsp70 chaperones in both the ADP- and ATP-bound states [41], [59]. Notably, a recent analysis of the multi-spanning cystic fibrosis transmembrane conductance regulator has shown that the release of TMs from the Sec61 translocon and their membrane integration is ATP-dependent [60], although – thus far – there are no known core translocon components that contain ATPase activity. Possibly, the Sec63/BiP participation may provide the missing link.

## Materials and Methods

### Plasmids

Human Sec62, Sec63, ERdj1, ERdj4, and BiP were obtained as full-length cDNA clones from Imagenes. For N-terminal tagging with the Myc epitope, Sec62 and Sec63 were cloned into pCMV-Myc (BD Biosciences, Clontech). The Sec63ΔHPD and Sec63ΔC mutants were created by mutagenesis using the QuikChange® II XL Site-Directed Mutagenesis Kit (Stratagene). Sec63ΔHPD carries alanine substitutions of the amino acids (aa) HPD at positions 132 to 134, while Sec63ΔC contains a premature stop codon generated at aa position 444 thereby deleting the C-terminal domain (aa 444–760) of Sec63. ERdj1, ERdj4, and BiP were inserted into p3xFLAG-CMV-14 (Sigma-Aldrich) thereby giving raise to C-terminally FLAG-tagged constructs. All constructs were verified by sequencing and cloning details are available on request. Mammalian expression vectors carrying the wt or mutant HBV envelope genes have been described previously [22], [28]. Briefly, pNI2.S.HA (HBV.S) and pNI2.L.HA (HBV.L) encode the S or the L gene, respectively, with a C-terminally fused HA epitope under the transcriptional control of the hMTIIa promoter. The HBV.SΔTM1 mutant lacks the first TM segment of the S protein, while the HBV.Ile9::L mutant carries the L gene with a foreign N-terminal signal sequence derived from human interleukin-9 [27], [28]. The bicistronic plasmid pCDNA/HA.Hip-L (HBV.L/Hip) encodes the HA-tagged L gene and the HA-tagged human Hip gene under the control of the hMTIIa or CMV promoter, respectively [22]. The M envelope gene of MHV strain A59, present in plasmid pTZ19R-M [37], was a gift from P. J. M. Rottier (Utrecht University, The Netherlands) and was subcloned into pCMV.HA (BD Biosciences, Clontech) for C-terminal tagging with the HA epitope (MHV.M). Plasmid pCI-VSVG expresses the G glycoprotein of VSV under control of the CMV promoter [35] and was obtained from G. Nolan (Stanford University School of Medicine, USA) as “Addgene plasmid 1733”. Plasmid pEYFP-ER encodes YFP with an N-terminal ER targeting sequence of calreticulin and a C-terminal ER retrieval sequence (BD Biosciences, Clontech). Plasmid pEYFP-Golgi carries a fusion gene consisting of YFP preceded by a N-terminal 81 aa sequence of human β1,4-galactosyltransferase (BD Biosciences, Clontech). The construct for the human HA-tagged hemopexin (Hxp) has been described [61]. Vectors encoding human CD63 with an N-terminal HA-tag or N-terminal EGFP fusion under control of the CMV promoter were gifts from G. Spoden (University Mainz, Germany). For EGFP tagging, the open reading frame of CD63 encoding aa 2–236 was fused in frame to aa 236 of EGFP (CD63.GFP) present in plasmid pEGFP-C1 (BD Biosciences, Clontech).

### siRNAs

To inhibit expression of Sec63, siRNA duplexes targeting the nucleotide positions 1942 to 1960 of Sec63 (CAAGAATGGTGGTGGCTTT) or esiRNA were used (Sigma-Aldrich). As a control, a nonsense siRNA with no known homology to mammalian genes was used (Quiagen).

**Table 1:** List of primers and hydrolysis probes used in qPCR.

Oligo name	Sequence
HBV.S-F	5′ TGTCCTCCAACTTGTCCTGGTT 3′
HBV.S-R	5′ AGGCATAGCAGCAGGATGAAGA 3′
HBV.S-Probe	5′ FAM-ATCGCTGGATGTGTCTGCGGCGTT-BHQ1 3′
YFP.ER-F	5′ ACCACATGAAGCAGCACGACTT 3′
YFP.ER-R	5′ TTGTAGTTGCCGTCGTCCTTGA 3′
YFP.ER-Probe	5′ FAM-AAGTCCGCCATGCCCGAAGGCTACGT-BHQ1 3′
CD63-F	5′ CCCATACGATGTTCCAGATTACGC 3′
CD63-R	5′ TTTCATTCCTCCTTCCACCGCT 3′
CD63-Probe	5′ FAM-TTATGGCCATGGAGGCCCGAATTCGGT-BHQ1 3′
VSV.G-F	5′ TCAAGCAGACGGTTGGATGTGT 3′
VSV.G-R	5′ TGAAGGATCGGATGGACTGTGT 3′
VSV.G-Probe	5′ FAM-ACCAGCGGAAATCACAAGTAGTGACCC-BHQ1 3′

### Antibodies

Commercially available antibodies were as follows: mouse anti-β-actin (Sigma-Aldrich), rabbit anti-calnexin (Santa Cruz Biotechnology), mouse anti-FLAG (Sigma-Aldrich), mouse anti-GFP (BD Biosciences, Clontech), mouse anti-HA (BAbCO), rat anti-HA (Roche), mouse anti-Myc (BAbCO), rabbit antibody against human Sec63 (Sigma-Aldrich), and mouse anti-VSV-G (Sigma-Aldrich). Peroxidase-labeled, secondary antibodies were obtained from Dianova, and fluorophor-labeled antibodies were purchased from Molecular Probes.

### Cell Culture and Transfection

Transfections of the human hepatocellular carcinoma cell line HuH-7 with plasmid DNAs were performed with LipoFectamine™ Plus (Invitrogen), while human embryonic kidney 293T cells were transfected with LipoFectamine™ 2000 (Invitrogen). The ratios of plasmid DNA used in (co)transfection experiments are indicated in the figure legends. For transfection of cells with siRNAs, the LipoFectamine™ RNAiMAX transfection reagent (Invitrogen) was used. Briefly, 5×10^5^ cells per well of a 6-well plate were transfected with 90 pmol siRNA or 200 ng esiRNA according to the protocol of the supplier. After 48 h, cells were retransfected with plasmid DNA using LipoFectamine™ Plus/2000 and harvested after additional 48 h. For proteasomal inhibition, cells were treated with 1.5 µM epoxomicin (Calbiochem) for 16 h prior to cell harvest.

### Cell Lysis and Protein Analysis

To probe for protein expression, cells were lysed with either the non-denaturing detergent Nonidet P-40 (NP-40) or the denaturing reagent sodium dodecylsulfate (SDS). NP-40 lysates were prepared by incubating the cells with Tris-buffered saline (50 mM Tris-HCl pH 7.5/150 mM NaCl) containing 0.5% NP-40 for 20 min on ice. Thereafter, lysates were centrifuged for 5 min at 13 000 *g* and 4°C. For lysis with SDS, the cells were scraped from the plates using 1 × Laemmli buffer, and cell suspensions were boiled for 10 min prior to centrifugation. Cell extracts were subjected to SDS-PAGE and Western blotting analyses using standard procedures. Immunoreactivity was determined by enhanced chemiluminescence (Western Lightning® Plus–ECL, PerkinElmer) and recorded on Amersham Hyperfilm ECL (GE Healthcare). The densitometric analysis of Western blot band intensities was based on linear, 8-bit, gray scale, transmitted light TIFF scans of the exposed films and was performed by using IMAGEQUANT® software (Molecular Dynamics). In parallel, cell culture media was harvested, cleared by centrifugation, and assayed for the secretion/release of proteins. The expression and secretion of the HBV.S envelope protein was measured using the Murex HBsAg Version 3 ELISA (Abbott). To evaluate the presence of damage and toxicity of transfected cells, lactate dehydrogenase (LDH) activity was determined in culture media using a colorimetric quantification assay (Roche).

### Trypsin Protection Assay

Microsomes were prepared from homogenized cells and subjected to a trypsin protection assay as described [22]. Briefly, microsomes were proteolyzed with trypsin in the presence or absence of 0.5% NP-40 for 60 min on ice. After inactivation of trypsin with aprotinin, solubilized samples were subjected to SDS-PAGE and immunoblotting.

### Fluorescence Microscopy

For immunostaining, cells grown on cover-slips were fixed and permeabilized with ice-cold methanol containing 2 mM EGTA. Cells were blocked in PBS containing 2% animal serum, incubated with the indicated primary antibodies for 1 h at 37°C, rinsed with PBS, and then incubated with AlexaFluor-tagged secondary antibodies for 1 h at 37°C. DNA was stained with Hoechst 33342 (Sigma-Aldrich). Z-stack images were acquired separately for each channel using a Zeiss Axiovert 200 M microscope equipped with a Plan-Apochromat 100× (1.4 NA) and a Zeiss Axiocam digital camera and were optically deconvoluted using the software supplied by Zeiss. Phase contrast images were obtained with the same microscope using phase contrast optics. For quantitative immunofluorescence analyses, images of randomly selected cells (about 100 cells per coverslip; *n* = 5) were collected at 100× magnification with identical settings.

### Real-time Quantitative PCR (qPCR) Analysis of Transcription

The mRNA of cotransfected HuH-7 cells was isolated using the Dynabeads® mRNA DIRECT™ Kit (Life Technologies) according to the Mini-scale guidelines. To eliminate trace amounts of genomic and plasmid DNA, the mRNA was treated with 5 U DNase I (Roche Applied Science) at 30°C for 20 minutes. Reverse transcription was performed with the Transcriptor Universal cDNA Master Kit (Roche Applied Science) and the cDNA was diluted 1∶3. The mRNA levels were determined by a TaqMan-chemistry based quantitative real-time PCR as absolute quantification using the standard curve method. Therefore, serial dilutions of plasmid DNA containing the respective target DNA (range 1×10^6^ to 1×10^−1^ fg/µl) were amplified in parallel. The PCR was performed with a 7300 Real-Time PCR System and the Sequence Detection Software 4.0 (Life Technologies). Each PCR reaction contained 4 µl aqua bidest, 0.4 µl of each primer (100 µM), 0.2 µl of each TaqMan probe (100 µM), 12.5 µl TaqMan® Gene Expression Master Mix (Life Technologies) and 7.5 µl cDNA. Primers and probes are listed in Table 1. The PCR reaction was initiated by a single step at 50°C for 2 minutes and 95°C for 10 minutes followed by 38 cycles at 95°C for 15 seconds and at 60°C for 60 seconds. Amplifications of samples and standard curves were performed in duplicate. The calculated data were converted into percentages whereupon the mean value of the control samples was set to 100%.
